# The impact of hormone replacement therapy on cardiovascular health in postmenopausal women: a narrative review

**DOI:** 10.3389/frph.2026.1745210

**Published:** 2026-01-22

**Authors:** Hafsah Tajammul Khalifey, Rutaba Mahereen, Raghda Adwan, Rafah Chahine, Marwa Kaidali, Samra Farhat Mirza, Sarah Noor Tullah, Saniyah Shaikh, Shahad Hammad, Hatouf H. Sukkarieh

**Affiliations:** College of Medicine, Alfaisal University, Riyadh, Saudi Arabia

**Keywords:** cardiovascular disease, cardiovascular health, estrogen, hormone replacement therapy, menopause, postmenopausal women, timing hypothesis

## Abstract

Menopause represents a pivotal transition in women's health, characterized by loss of ovarian hormone production and substantially increased cardiovascular disease (CVD) risk. Hormone replacement therapy (HRT), once widely prescribed for both symptom management and cardiovascular protection, faced significant scrutiny following the Women's Health Initiative (WHI) trial, which associated conventional regimens with elevated risks of stroke, thromboembolism, and breast cancer. Contemporary evidence demonstrates that cardiovascular outcomes vary considerably based on formulation, route of administration, timing of initiation, and patient-specific factors. Modern strategies emphasize individualized patient selection, lower-dose regimens, and transdermal delivery methods. The “timing hypothesis” proposes that HRT initiated within 10 years of menopause onset or before age 60 may confer cardiovascular benefit, whereas later initiation may increase cardiovascular risk. This narrative review synthesizes historical and contemporary evidence on HRT and CVD, examines mechanistic pathways including vascular, metabolic, and immunomodulatory effects, and evaluates evolving clinical guidelines. Despite substantial progress, significant uncertainties persist due to trial heterogeneity, underrepresentation of diverse populations, and inconsistent long-term outcomes. Future research priorities include personalized therapeutic approaches, mechanistic investigations, and rigorous evaluation of cardiovascular endpoints to definitively establish HRT's role in preventive cardiology. This review provides updated evidence for clinicians navigating complex decisions regarding HRT use in postmenopausal women, with emphasis on cardiovascular risk stratification and individualized treatment planning.

## 1 Introduction

Menopause is a natural biological transition that now occupies approximately one-third of women's lives due to increasing longevity. The World Health Organization defines menopause as “the permanent cessation of menstruation resulting from loss of ovarian follicular activity” (1). Natural menopause is confirmed after 12 consecutive months of amenorrhea without other identifiable physiological or pathological causes. Because this endocrine transition is accompanied by a marked rise in cardiovascular risk, menopause has become a key inflection point for evaluating preventive strategies, including hormone replacement therapy.

The hormonal cascade during menopausal transition begins with declining ovarian inhibin production, resulting in elevated follicle-stimulating hormone (FSH) levels. Subsequently, irregular and progressively reduced estradiol levels emerge, driving characteristic vasomotor symptoms and significant cardiovascular changes. Vasomotor symptoms (VMS) are strongly associated with vascular instability and represent the most common indication for hormone replacement therapy (HRT). Additionally, sleep disturbances, mood alterations, and urogenital symptoms including vaginal atrophy and urinary complaints substantially diminish quality of life. Critically, the presence of VMS correlates with increased cardiovascular risk, highlighting the interconnection between symptomatic manifestations and long-term cardiovascular disease (CVD) risk (2, 3). This risk intensifies after age 50, with the proportion of women at intermediate-to-high cardiovascular risk nearly doubling between ages 51 and 55 (4). This dual relevance (addressing both symptom burden and cardiovascular risk) helps explain why HRT has remained central to menopausal care and a continuing focus of cardiovascular evaluation.

HRT has been prescribed for decades to manage menopausal symptoms, with historical assumptions regarding cardiovascular protection. Women with intact uteri receive combination therapy (estrogen plus progestin) to prevent endometrial hyperplasia and malignancy, whereas hysterectomized women are prescribed estrogen-only regimens. Multiple formulations varying in estrogen and progestogen types and potencies are available, delivered via oral, transdermal, or vaginal routes, highlighting both therapeutic flexibility and prescribing complexity (5).

The evolution of HRT has been marked by a transition from the aforementioned standardized “conventional” approaches to more individualized “modern” strategies. Conventional HRT, using fixed formulations such as conjugated equine estrogens (CEE) combined with medroxyprogesterone acetate (MPA), dominated late 20th-century practice and formed the basis for major trials, including the Women's Health Initiative (WHI) (6). These regimens were criticized for their uniform dosing and limited flexibility (7). In contrast, modern HRT emphasizes personalized approaches, incorporating lower doses, alternative delivery routes (transdermal or vaginal), bioidentical hormones, and treatment tailored to individual cardiovascular risk profiles, symptom burden, and patient preferences (8). This shift from standardized therapy to personalized care reflects efforts to optimize symptom relief while minimizing risks, particularly thromboembolic and cardiovascular events (9).

HRT's historical trajectory spans initial estrogen monotherapy in the mid-20th century, gaining prominence following publication of “Feminine Forever” in the 1960s. The addition of progestin in the 1970s addressed endometrial cancer concerns (10, 11), and widespread adoption in the 1990s for presumed cardiovascular and bone benefits. However, dramatic prescription reduction following WHI trial results in the 2000s revealing increased risks in older women. Subsequent validation of the “timing hypothesis” demonstrating superior benefit-risk profiles with early initiation (12, 13).

This narrative review aims to synthesize current evidence regarding HRT's benefits and risks, with specific focus on cardiovascular health implications. By distinguishing conventional from modern approaches and examining recent clinical and mechanistic data, this review elucidates HRT's evolving role in preventive cardiology for postmenopausal women.

## 2 Methodology

### 2.1 Review design and objectives

This narrative review was designed to explore and synthesize evidence regarding the impact of hormone replacement therapy on cardiovascular health in postmenopausal women, encompassing both historical perspectives and contemporary data. The review aimed to differentiate cardiovascular effects of conventional vs. modern HRT regimens and provide clinically relevant guidance for practitioners.

### 2.2 Population and interventions

The target population comprised postmenopausal women (defined as ≥12 months of amenorrhea) receiving HRT. Interventions examined included estrogen-only therapy and combined estrogen-progestin therapy, delivered via oral, transdermal, or vaginal routes. Studies comparing conventional regimens (CEE with MPA) to modern approaches (transdermal estradiol, micronized progesterone) were prioritized.

### 2.3 Outcomes of interest

Primary cardiovascular outcomes examined included coronary heart disease, myocardial infarction, stroke, venous thromboembolism, and atherosclerosis progression. Secondary outcomes encompassed surrogate cardiovascular markers including lipid profiles, endothelial function, insulin sensitivity, and inflammatory biomarkers.

### 2.4 Literature search strategy

A comprehensive literature search was conducted using PubMed and Google Scholar databases. The search strategy employed the following combination: {[Hormone replacement therapy(Title)] AND [Cardiovascular(Title)]} AND [menopause(Title/Abstract)]. This title-specific search strategy was chosen to prioritize high-relevance publications where HRT and cardiovascular health were the primary focus, ensuring a concentrated synthesis of the most pertinent evidence. Only English-language publications were included. No date restrictions were applied to capture historical context, though emphasis was placed on studies published from 2000 to 2025. The search was conducted in October 2024.

### 2.5 Inclusion and exclusion criteria

Inclusion criteria encompassed: (1) studies involving postmenopausal women; (2) interventions including any form of HRT; (3) cardiovascular outcomes or biomarkers reported; (4) randomized controlled trials, cohort studies, meta-analyses, and systematic reviews; (5) English language publications.

Exclusion criteria included: (1) studies exclusively addressing non-cardiovascular outcomes (osteoporosis, cognitive function, cancer) without cardiovascular data; (2) studies focusing solely on premenopausal or perimenopausal populations; (3) animal or *in vitro* studies without clinical correlation; (4) studies lacking adequate description of HRT type, timing, or dosing; (5) studies focused exclusively on awareness, knowledge, or perception without health outcomes data.

### 2.6 Limitations

This narrative review has several limitations. The literature search was limited to two databases, potentially missing relevant studies indexed exclusively in other databases such as Embase, Web of Science, or Cochrane Library. Google Scholar, while comprehensive, is not typically considered a primary academic database for systematic reviews. No formal quality assessment or risk of bias evaluation was conducted. Publication bias and heterogeneity in study designs, populations, HRT formulations, and outcome measures limit direct comparisons. As a narrative rather than systematic review, formal evidence grading was not performed. These limitations should be considered when interpreting the synthesized evidence. Furthermore, although not systematic, the review adheres to good-practice narrative review methodology by providing a transparent and critical synthesis of the available evidence.

## 3 Mechanisms of cardiovascular action

**Figure 1:**
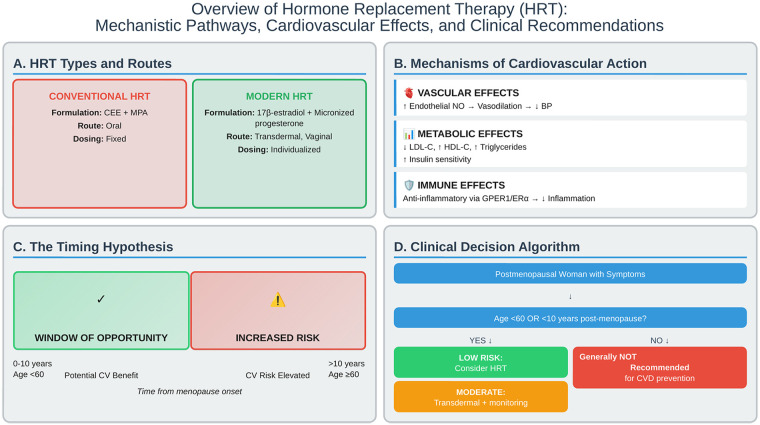
Comprehensive overview of hormone replacement therapy (HRT) and cardiovascular health. **(A)** Comparison of conventional and modern HRT formulations and delivery routes. **(B)** Primary mechanisms of cardiovascular action including vascular, metabolic, and immune pathways. **(C)** The timing hypothesis illustrates the window of opportunity for cardiovascular benefit. **(D)** Clinical decision-making algorithm for HRT initiation based on age, time since menopause, and cardiovascular risk profile. BP, blood pressure; CEE, conjugated equine estrogen; CV, cardiovascular; ERα, estrogen receptor alpha; GPER1, G-protein-coupled estrogen receptor; HDL-C, high-density lipoprotein cholesterol; HRT, hormone replacement therapy; LDL-C, low-density lipoprotein cholesterol; MPA, medroxyprogesterone acetate; NO, nitric oxide.

Understanding the biological mechanisms through which HRT influences cardiovascular health is essential for optimizing therapeutic strategies and predicting individual responses. Estrogen and progesterone exert multifaceted effects on the cardiovascular system through vascular, metabolic, and immunomodulatory pathways (Figure 1).

### 3.1 Vascular effects

Estrogen exerts profound effects on the cardiovascular system through multiple vascular mechanisms. Endothelial dysfunction is a marked event in atherosclerosis development and a strong predictor of prognostic cardiovascular events. By estrogen-mediated upregulation of endothelial nitric oxide production and suppression of endothelium-derived contracting factors, endothelial dysfunction may be prevented and even reversed (14). 17β-estradiol promotes vascular relaxation through direct effects on vascular smooth muscle and by stimulating endothelial nitric oxide synthase activity, functioning as a potent vasodilator and hypotensive agent (15). Progesterone enhances these effects through natriuretic actions, blood pressure reduction, and coronary vasodilation. Both nuclear and membrane receptors mediate rapid activation of secondary messenger pathways in vascular cells, modulating calcium influx and enhancing endothelial nitric oxide synthase activity (16). Collectively, these mechanisms contribute to vascular stability and cardioprotection.

### 3.2 Metabolic effects

HRT significantly influences lipid metabolism and glucose homeostasis. A randomized clinical trial evaluating HRT effects on plasma atherogenicity in postmenopausal women demonstrated that at three months, HRT significantly reduced plasma levels of lipoprotein(a) [Lp(a)], apolipoprotein B (Apo B), total cholesterol (TC), low-density lipoprotein cholesterol (LDL-C), and TC/HDL-C and LDL-C/HDL-C ratios compared to placebo (17). Cross-sectional data confirmed that postmenopausal women not receiving HRT exhibited significantly higher Lp(a), LDL-C, and plasma cholesterol levels compared to both premenopausal women and postmenopausal women receiving HRT. However, plasma triglyceride levels were notably elevated in women receiving HRT compared to both comparison groups. Additionally, HRT did not significantly alter oxidized LDL levels, suggesting neutral effects on LDL oxidation (18).

Beyond lipid modulation, estrogen therapy improves insulin sensitivity. In women with type 2 diabetes, HRT improved glucose management and metabolic parameters, reducing overall cardiovascular risk and mitigating metabolic syndrome components (19).

### 3.3 Immunomodulatory and inflammatory effects

Estrogen influences immunological and inflammatory pathways relevant to cardiovascular pathophysiology. Estradiol promotes anti-inflammatory responses by modulating pro-inflammatory signaling through the G-protein-coupled estrogen receptor (GPER1) and estrogen receptor alpha (ERα) (20). These combined vascular, metabolic, and anti-inflammatory effects may collectively improve metabolic syndrome components and potentially reduce cardiovascular event risk.

## 4 Clinical evidence from major trials

The cardiovascular effects of HRT have been extensively investigated through landmark clinical trials that have profoundly influenced clinical practice guidelines. Understanding the evolution of this evidence is crucial for contextualizing current recommendations.

### 4.1 The women's health initiative (WHI)

The WHI trial, initiated in 1993, strongly reshaped clinical practice and remains the reference point for risk communication; it represents the largest randomized controlled trial examining HRT effects on chronic disease outcomes. The study enrolled over 27,000 postmenopausal women aged 50–79 years and consisted of two arms: estrogen plus progestin (CEE 0.625 mg/day with MPA 2.5 mg/day) for women with intact uteri, and estrogen-only therapy for hysterectomized women (21, 22). The combined therapy arm was prematurely terminated in 2002 after a mean follow-up of 5.2 years due to increased risks of invasive breast cancer, coronary heart disease, stroke, and pulmonary embolism, despite reductions in hip fractures and colorectal cancer (21). The estrogen-only arm continued until 2004, demonstrating a more favorable cardiovascular profile with no increase in coronary heart disease risk and a potential reduction in breast cancer incidence (22).

Critical analysis of WHI data revealed important age-related and timing-dependent effects. Subgroup analyses demonstrated that younger women (ages 50–59) or those within 10 years of menopause onset exhibited trends toward cardiovascular benefit, whereas older women or those more distant from menopause experienced increased cardiovascular risk (23). These findings gave rise to the “timing hypothesis” and fundamentally reshaped HRT prescribing practices.

### 4.2 The heart and estrogen/progestin replacement study (HERS)

The HERS trial, published in 1998, was the first large randomized controlled trial specifically designed to test whether HRT could prevent recurrent coronary events in postmenopausal women with established coronary disease (24). The study enrolled 2,763 women with documented coronary disease, randomizing them to CEE plus MPA or placebo. After an average follow-up of 4.1 years, HRT did not reduce the overall rate of coronary events. Notably, the study revealed a pattern of early increased risk followed by potential benefit in later years, with significantly more coronary events in the HRT group during the first year (24). The HERS II follow-up study extended observation through 6.8 years and confirmed no overall cardiovascular benefit (25), establishing that HRT should not be initiated for secondary prevention of cardiovascular disease.

### 4.3 The early versus late intervention trial with estradiol (ELITE)

The ELITE trial directly tested the timing hypothesis by comparing cardiovascular effects of oral 17β-estradiol in early postmenopausal women (<6 years since menopause) vs. late postmenopausal women (≥10 years since menopause) (26). The primary outcome was rate of change in carotid artery intima-media thickness (CIMT), a validated surrogate marker for atherosclerosis progression. After a median follow-up of 5 years, estradiol significantly reduced CIMT progression in early postmenopausal women but showed no effect in late postmenopausal women (26). This landmark trial provided direct mechanistic evidence supporting age-dependent and timing-dependent cardiovascular effects of HRT.

### 4.4 Danish osteoporosis prevention study (DOPS)

The DOPS trial examined long-term cardiovascular outcomes in recently menopausal women randomized to HRT or no treatment (27). Unlike WHI, DOPS enrolled younger women (mean age 50 years) who were recently menopausal. After 10 years of treatment and an additional 6 years of follow-up, women randomized to HRT demonstrated significant reductions in mortality, heart failure, and myocardial infarction without increased cancer, venous thromboembolism, or stroke (27, 28). These findings strongly supported the timing hypothesis and demonstrated that HRT initiated early in menopause may provide cardiovascular protection.

Table 1 summarizes the key characteristics and findings from these major clinical trials, highlighting the evolution of evidence regarding HRT and cardiovascular outcomes.

**Table 1:** Summary of major clinical trials examining HRT and cardiovascular outcomes.

Trial	*N*	Population	Intervention	Follow-up	Key cardiovascular findings
WHI E + P (2002)	16,608	Postmenopausal women 50–79 years with intact uterus	CEE 0.625 mg/day + MPA 2.5 mg/day	5.2 years (median)	↑ coronary heart disease, stroke, venous thromboembolism; age-stratified analysis showed trend toward benefit in women 50–59 years
WHI E-only (2004)	10,739	Postmenopausal women 50–79 years post-hysterectomy	CEE 0.625 mg/day	6.8 years (median)	No ↑ coronary heart disease; trend toward ↓ myocardial infarction in younger women; ↑ stroke
HERS (1998)	2,763	Postmenopausal women with established CHD	CEE 0.625 mg/day + MPA 2.5 mg/day	4.1 years (mean)	No overall benefit; early ↑ risk (year 1), potential late benefit; not for secondary prevention
ELITE (2016)	643	Early (<6 yrs) vs. late (≥10 yrs) postmenopausal women	Oral 17β-estradiol 1 mg/day ± progesterone gel	5 years (median)	↓ CIMT progression in early but not late postmenopausal women; supports timing hypothesis
DOPS (2012)	1,006	Recently menopausal women (mean age 50 yrs)	Estradiol ± norethisterone acetate	10 years treatment + 6 years follow-up	↓ mortality, heart failure, myocardial infarction; no ↑ cancer, venous thromboembolism, or stroke; supports early initiation

CEE, conjugated equine estrogens; CIMT, carotid intima-media thickness; DOPS, Danish Osteoporosis Prevention Study; E + P, estrogen plus progestin; ELITE, Early Versus Late Intervention Trial with Estradiol; HERS, Heart and Estrogen/Progestin Replacement Study; MPA, medroxyprogesterone acetate; WHI, Women's Health Initiative; ↑, increased; ↓, decreased.

## 5 The timing hypothesis: clinical application

The “timing hypothesis” or “window of opportunity” concept has emerged as a central paradigm in understanding HRT's cardiovascular effects. This hypothesis proposes that cardiovascular outcomes depend critically on the timing of HRT initiation relative to menopause onset and underlying vascular health status (29).

### 5.1 Biological basis of the timing hypothesis

The mechanistic foundation for timing-dependent effects relates to progressive atherosclerotic changes that occur with advancing age and years since menopause. In younger postmenopausal women with relatively healthy arteries, estrogen promotes endothelial function, inhibits smooth muscle proliferation, and prevents early atherosclerotic lesion formation (30). However, in older women with established atherosclerosis, estrogen may destabilize existing plaques, increase inflammation within atherosclerotic lesions, and promote thrombotic events (31). Mechanistically, the “window of opportunity” is defined by the transition from healthy endothelium to established atherosclerosis. In the younger ELITE cohort, high estrogen receptor alpha (ERα) expression and stable vascular smooth muscle allow for beneficial nitric oxide production. Conversely, in the older WHI populations, age-related ERα downregulation and established plaques may lead to estrogen-induced pro-inflammatory signaling and plaque destabilization, explaining the observed increase in cardiovascular events.

### 5.2 Evidence supporting the window of opportunity

Multiple lines of evidence support the timing hypothesis. *Post-hoc* analyses of WHI data stratified by age and time since menopause demonstrated markedly different cardiovascular outcomes. Women who initiated HRT within 10 years of menopause or before age 60 showed trends toward reduced coronary heart disease (hazard ratio (HR): 0.76, 95% CI: 0.50–1.16 for estrogen plus progestin; HR: 0.59, 95% CI: 0.38–0.90 for estrogen-only therapy), whereas women initiating HRT more than 20 years after menopause or at age 70 or older experienced increased coronary risk (23, 32). The ELITE trial provided direct mechanistic evidence by demonstrating that 17β-estradiol reduced atherosclerosis progression in early but not late postmenopausal women (26). Long-term follow-up of the DOPS cohort confirmed that HRT initiated in recently menopausal women reduced cardiovascular events and mortality over 16 years of follow-up (27, 28).

### 5.3 Clinical implications for practice

The timing hypothesis has profoundly influenced clinical practice guidelines. Current recommendations emphasize that HRT may be considered for women within 10 years of menopause onset or before age 60 if vasomotor symptoms are present (given there are no contraindications). On the other hand, HRT initiation for the purpose of disease prevention is generally not recommended for women past 10 years of menopause onset or above 60 years of age (33, 34). Individualized risk assessment considering cardiovascular risk factors, thrombotic risk, breast cancer risk, and symptom severity guides therapeutic decision-making.

## 6 Safety considerations and risk stratification

While HRT initiated during the window of opportunity may provide cardiovascular benefit for appropriately selected women, comprehensive safety evaluation remains essential. Thrombotic risk, stroke risk, and breast cancer risk require careful assessment.

### 6.1 Venous thromboembolism risk

Oral estrogen consistently increases venous thromboembolism (VTE) risk approximately twofold, attributed to hepatic first-pass metabolism inducing procoagulant changes, translating to a baseline VTE risk of ∼1 per 1,000 woman-years and an estimated additional ∼1.5 VTE events per 1,000 women per year (NNH ≈ 667 per year), with the highest relative risk observed during the first year of therapy (35, 36). Transdermal estrogen appears safer, with multiple observational studies demonstrating no significant VTE risk increase (37, 38). For women with elevated thrombotic risk (obesity, thrombophilia, limited mobility, previous VTE), transdermal estrogen delivery is strongly preferred. Absolute VTE risk remains low in younger postmenopausal women without risk factors, but increases substantially with advancing age and comorbidities.

### 6.2 Stroke risk

Stroke risk varies by estrogen type, dose, and route of administration. Oral estrogen increases ischemic stroke risk, particularly at higher doses, corresponding to a baseline stroke rate of approximately 2.85 per 1,000 women per year and an estimated additional ∼0.8 strokes per 1,000 women per year (rate ratio ∼1.28; approximate NNH ≈ 1,250 per year) (39). Lower-dose regimens and transdermal delivery appear safer. Low-dose transdermal patches (≤50 μg) have not been associated with an increased stroke risk, whereas higher-dose patches (>50 μg) are associated with increased risk (39). The absolute increase in stroke risk is small in younger postmenopausal women but becomes clinically significant in older women with elevated baseline cardiovascular risk. Hypertension control and cardiovascular risk factor management are essential before HRT initiation.

### 6.3 Breast cancer considerations

Breast cancer risk varies by HRT formulation and duration. Estrogen-only therapy shows minimal breast cancer risk increase with up to 7 years of use (22). Estrogen plus progestin increases breast cancer risk, with synthetic progestins (particularly medroxyprogesterone acetate) conferring higher risk than micronized progesterone (40, 41). This relative increase corresponds to approximately 9 additional cases of invasive breast cancer per 10,000 women per year during the intervention phase of combined estrogen–progestin therapy (22). Baseline breast cancer risk assessment, regular screening, and consideration of the lowest effective dose for the shortest duration are essential components of individualized risk–benefit evaluation.

### 6.4 Cardiovascular risk stratification

Comprehensive cardiovascular risk assessment should precede HRT initiation. Traditional cardiovascular risk factors including hypertension, diabetes, dyslipidemia, smoking, obesity, and family history require evaluation. Women at elevated cardiovascular risk may benefit from transdermal estrogen delivery, lower doses, and intensive cardiovascular risk factor management. Table 2 provides a framework for risk stratification and HRT selection based on individual patient characteristics.

**Table 2:** Risk stratification and HRT recommendations for postmenopausal women.

Clinical scenario	Cardiovascular risk	Preferred HRT approach	Monitoring requirements
Age <60 or <10 years postmenopause; no cardiovascular risk factors	Low	Oral or transdermal estrogen; micronized progesterone if uterus intact	Annual BP, lipids; standard breast/cervical screening
Age <60 or <10 years postmenopause; 1–2 cardiovascular risk factors (HTN, dyslipidemia, obesity)	Moderate	Transdermal estrogen preferred; low dose; micronized progesterone	Intensive cardiovascular risk factor management; BP, lipids, glucose every 3–6 months
Age <60 or <10 years postmenopause; ≥ 3 cardiovascular risk factors or diabetes	High	Transdermal low-dose estrogen; consider non-hormonal alternatives; shared decision-making	Aggressive cardiovascular risk factor management; frequent monitoring; cardiology consultation if indicated
Age ≥60 or >10 years postmenopause; established CVD	Very High	HRT generally not recommended for CVD prevention; consider non-hormonal alternatives for symptoms	Standard CVD management; if HRT continued for symptoms, close monitoring required
History of VTE or thrombophilia	High thrombotic risk	Oral HRT contraindicated; transdermal low-dose may be considered with hematology consultation	Close monitoring; patient education on venous thromboembolism symptoms; consider thromboprophylaxis for high-risk situations

BP, blood pressure; CVD, cardiovascular disease; HRT, hormone replacement therapy; HTN, hypertension;.

## 7 Modern HRT formulations and delivery routes

Contemporary HRT strategies emphasize individualized selection of hormone type, dose, and delivery route to optimize efficacy while minimizing risks. Understanding differences between conventional and modern formulations is essential for evidence-based prescribing.

### 7.1 Estrogen formulations

Conventional HRT predominantly utilized conjugated equine estrogens (CEE), derived from pregnant mare urine and containing multiple estrogen compounds. Modern approaches favor bioidentical 17β-estradiol, which more closely mimics endogenous human estrogen and may provide a more favorable safety profile (42). Oral estradiol undergoes hepatic first-pass metabolism, producing high concentrations of estrone and metabolites that influence hepatic protein synthesis, potentially increasing thrombotic risk. Transdermal estradiol bypasses hepatic first-pass metabolism, maintaining more physiologic estradiol-to-estrone ratios and avoiding adverse hepatic effects (43). Observational studies consistently demonstrate lower VTE and potentially lower stroke risk with transdermal vs. oral estrogen delivery (37, 38, 44).

### 7.2 Progestogen selection

For women with intact uteri, progestogen is essential to prevent endometrial hyperplasia. Conventional regimens utilized synthetic progestins such as medroxyprogesterone acetate (MPA), which demonstrated adverse metabolic and cardiovascular effects in clinical trials (21). Modern approaches increasingly favor micronized progesterone, due to its close resemblance to endogenous progesterone and metabolic neutrality, and potentially lower risk of breast cancer (40, 41). Alternative progestins including dydrogesterone and norethisterone acetate offer intermediate profiles. Levonorgestrel-releasing intrauterine systems provide effective endometrial protection with minimal systemic progestogen exposure (45).

### 7.3 Dosing strategies

Contemporary practice emphasizes the lowest effective dose for symptom control. Lower estrogen doses (e.g., estradiol 0.5–1 mg orally or 25–50 mcg transdermally) provide adequate symptom relief for many women while potentially reducing adverse event risks (46). Ultra-low-dose vaginal estrogen preparations effectively treat genitourinary syndrome of menopause with minimal systemic absorption (47). Individual dose titration based on symptom response and tolerability optimizes outcomes.

Table 3 compares conventional and modern HRT regimens, highlighting key differences in formulation, delivery, and clinical characteristics.

**Table 3:** Comparison of conventional and modern HRT regimens.

Characteristic	Conventional HRT	Modern HRT
Estrogen Type	Conjugated equine estrogens (CEE)	Bioidentical 17β-estradiol
Progestogen	Medroxyprogesterone acetate (MPA)	Micronized progesterone, dydrogesterone, or LNG-IUS
Primary Route	Oral	Transdermal (patch, gel) or vaginal; oral bioidentical estradiol
Dosing Strategy	Fixed standard dose (CEE 0.625 mg + MPA 2.5 mg)	Individualized; lowest effective dose (e.g., estradiol 0.5–1 mg oral or 25–50 mcg transdermal)
Hepatic First-Pass	Significant; alters hepatic protein synthesis	Avoided with transdermal delivery; minimal with low-dose oral
VTE Risk	Increased (∼2-fold with oral CEE)	Lower with transdermal estradiol; minimal VTE risk increase
Breast Cancer Risk	Increased with CEE + MPA (WHI: ∼8 additional cases per 10,000 person-years)	Lower with estradiol + micronized progesterone; estrogen-only shows minimal risk
Personalization	One-size-fits-all approach; limited flexibility	Tailored to individual CV risk, symptoms, preferences; multiple formulation and dose options

These distinctions between conventional and modern HRT are consistent with current clinical guideline recommendations, including the North American Menopause Society (NAMS, 2022) (33) and International Menopause Society (IMS, 2023) (34) guidelines.

CEE, conjugated equine estrogens; CV, cardiovascular; HRT, hormone replacement therapy; LNG-IUS, levonorgestrel-releasing intrauterine system; MPA, medroxyprogesterone acetate; VTE, venous thromboembolism; WHI, Women's Health Initiative.

## 8 Cardiovascular risk in specific female populations

Female-specific reproductive histories which act as “risk enhancers” should be included in cardiovascular risk assessment for HRT in addition to regular considerations. Polycystic Ovary Syndrome (PCOS) and Hypertensive Disorders of Pregnancy (HDP), which includes preeclampsia, are linked to a markedly increased lifetime risk of CVD (12, 33).

### 8.1 Hypertensive disorders of pregnancy

Preeclamptic women have a three- to four-fold higher chance of developing chronic hypertension and ischemic heart disease in later life, as well as persistent vascular dysfunction. The “timing hypothesis” is especially pertinent to these women since early HRT initiation could help maintain endothelial function that was previously reduced during pregnancy (2). To reduce the effect on blood pressure and prevent the hepatic first-pass effect on coagulation factors, transdermal estradiol is the recommended delivery method for these patients (33).

### 8.2 Polycystic ovary syndrome (PCOS)

Lifelong metabolic problems, such as insulin resistance, dyslipidemia, and a higher incidence of metabolic syndrome, are characteristics of PCOS. Current research indicates that bioidentical HRT can enhance insulin sensitivity and glycemic control, despite prior findings raising concerns about HRT in metabolically high-risk women (2, 34). Specifically, findings in women with type 2 diabetes demonstrate that HRT can mitigate metabolic syndrome components, suggesting a potential cardioprotective role for PCOS patients transitioning into menopause (19).

Individualized risk stratification is crucial for these high-risk groups in order to strike a balance between long-term cardiovascular mitigation techniques and the symptomatic advantages of hormone replacement therapy.

## 9 Current clinical guidelines and recommendations

Multiple professional societies have developed evidence-based guidelines for HRT use in postmenopausal women. While recommendations share common principles, nuanced differences reflect evolving evidence and regional perspectives.

### 9.1 North American menopause society (NAMS)

NAMS 2022 position statement emphasizes that for women younger than 60 years or within 10 years of menopause onset with bothersome vasomotor symptoms and no contraindications, benefits of HRT generally outweigh risks (33). The society recommends individualized therapy selection and preferential use of transdermal estrogen for women at elevated VTE or cardiovascular risk. Additionally, it advocates for the use of micronized progesterone or progestin with the most favorable benefit-risk profile. NAMS advises against routine HRT use solely for chronic disease prevention but acknowledges potential cardiovascular and other benefits when initiated during the window of opportunity for appropriate candidates (33). Recent regulatory trends and updated therapeutic labeling emphasize the “lowest effective dose” for the shortest duration, focusing on individualized cardiovascular risk stratification as a prerequisite for initiation.

### 9.2 International menopause society (IMS)

IMS recommendations align closely with NAMS, emphasizing the importance of timing for cardiovascular effects (48). IMS highlights that observational studies and reanalysis of clinical trials support cardiovascular benefit or neutrality when HRT is initiated in recently menopausal women, contrary to the reports of increased risk in older women or those distant from menopause. IMS recommends transdermal estrogen for women with cardiovascular risk factors and encourages consideration of metabolic effects when selecting progestogens (48).

### 9.3 European society of cardiology (ESC)

ESC guidelines on cardiovascular disease prevention in women state that HRT should not be used for primary or secondary prevention of cardiovascular disease (49). However, ESC acknowledges that for symptomatic women within 10 years of menopause and at low cardiovascular risk, HRT may be used for symptom management without adversely affecting cardiovascular outcomes. ESC emphasizes comprehensive cardiovascular risk assessment, blood pressure control, and lipid management before HRT initiation (49).

### 9.4 Shared decision-making and patient-centered care

All guidelines emphasize individualized risk-benefit assessment and shared decision-making. Clinicians should discuss potential benefits (symptom relief, quality of life improvement, possible cardiovascular benefit if initiated early) and risks (VTE, stroke, breast cancer) in the context of individual patient characteristics, preferences, and values. Regular reassessment of continued HRT need and consideration of the lowest effective dose for the shortest duration consistent with treatment goals and benefits are recommended (33, 48, 49).

### 9.5 Regulatory landscape and modern guidelines

Modernizing risk communication for estrogen products has been the goal of recent regulatory changes. The FDA announced labeling changes in 2024–2025, eliminating nonspecific boxed warnings that generally connected HRT to CVD and cognitive decline (5). These warnings were primarily based on early, non-stratified WHI findings. The updated labeling emphasizes individualized assessment based on age, time since menopause, and route of administration, acknowledging that early initiation often confers a more favorable cardiovascular profile (33). This change is consistent with current guidelines for menopausal care, which emphasize shared, evidence-based conversations between clinicians and patients.

## 10 Future directions and research priorities

Despite substantial progress in understanding HRT's cardiovascular effects, important knowledge gaps persist. Future research should address several critical areas to refine clinical practice and optimize outcomes.

### 10.1 Precision medicine approaches

Identifying biomarkers and genetic predictors of HRT response could enable personalized therapeutic selection. Polymorphisms in estrogen receptor genes, metabolic enzymes, and coagulation factors may influence cardiovascular outcomes (50). Integration of genetic, metabolic, and clinical risk factors into precision medicine algorithms could identify women most likely to benefit from HRT while avoiding therapy in those at elevated risk. Prospective studies examining pharmacogenomic predictors of HRT response are needed.

### 10.2 Long-term cardiovascular outcomes

Extended follow-up of randomized trials and large prospective cohort studies are essential to definitively establish long-term cardiovascular effects of modern HRT formulations initiated during the window of opportunity. Studies should examine hard cardiovascular endpoints (myocardial infarction, stroke, cardiovascular mortality) rather than solely surrogate markers. Comparative effectiveness research evaluating different estrogen types, progestogens, doses, and delivery routes would inform optimal regimen selection (51).

### 10.3 Diverse and representative populations

Race, ethnicity, metabolic profiles, and socioeconomic status all have a significant impact on HRT response and cardiovascular risk. Black and white women have very different metabolomic profiles, according to research, and differences in lipid and amino acid metabolites are directly linked to an increased risk of coronary heart disease (52). Additionally, metabolic risk factors like obesity and hypertension are more common in racial and ethnic minorities, which may change the protective effects of HRT (49). Through biological and psychosocial pathways, socioeconomic status further complicates these outcomes; lower socioeconomic status is associated with higher mortality from CVD and less access to healthcare (14). To guarantee fair and culturally appropriate menopausal care, future research must incorporate genetic differences in drug metabolism and social determinants.

### 10.4 Novel therapeutic strategies

Emerging therapies including selective estrogen receptor modulators (SERMs), tissue-selective estrogen complexes (TSECs), and neurokinin-3 receptor antagonists offer alternatives or complements to traditional HRT (53). Comparative studies examining cardiovascular effects of these agents vs. bioidentical hormones would expand therapeutic options. Investigation of combination strategies integrating cardiovascular preventive therapies (statins, antihypertensives) with HRT may optimize cardiovascular outcomes in high-risk women.

### 10.5 Mechanistic investigations

Deeper understanding of molecular mechanisms underlying age-dependent and timing-dependent HRT effects would advance therapeutic development. Research examining estrogen receptor signaling, epigenetic modifications, inflammasome activation, and endothelial progenitor cell function in the context of aging and atherosclerosis may reveal novel therapeutic targets (54). Translational studies bridging basic science discoveries with clinical applications are essential.

## 11. Conclusions

The relationship between hormone replacement therapy and cardiovascular health in postmenopausal women is complex and timing-dependent. The evolution from conventional to modern HRT strategies reflects substantial progress in understanding mechanistic pathways, interpreting clinical trial evidence, and implementing individualized therapeutic approaches. The timing hypothesis—supported by multiple lines of evidence including landmark trials, mechanistic studies, and long-term observational data—establishes that cardiovascular effects depend critically on age, time since menopause, and underlying vascular health at HRT initiation.

For appropriately selected women younger than 60 years or within 10 years of menopause onset with bothersome vasomotor symptoms and no contraindications, HRT initiated during the window of opportunity may provide cardiovascular benefit or neutrality while effectively managing symptoms and improving quality of life. Modern formulations emphasizing bioidentical hormones, transdermal delivery, micronized progesterone, and individualized dosing offer improved safety profiles compared to conventional regimens. Outcomes can be optimized via comprehensive cardiovascular risk assessment, preference for transdermal estrogen in women with elevated cardiovascular or thrombotic risk, intensive risk factor management, and regular monitoring.

Conversely, HRT should not be initiated in women aged 60 years or older or more than 10 years past menopause onset for the primary purpose of cardiovascular disease prevention. For these women, non-hormonal symptom management strategies should be prioritized, and if HRT is considered for refractory symptoms, comprehensive shared decision-making regarding risks and benefits is essential.

Important knowledge gaps persist, particularly regarding long-term cardiovascular outcomes with modern HRT formulations, optimal therapeutic strategies for diverse populations, precision medicine approaches incorporating genetic and biomarker predictors, and comparative effectiveness of emerging alternatives. Continued research addressing these priorities will further refine evidence-based recommendations and optimize cardiovascular health outcomes for postmenopausal women.

This narrative review synthesizes current evidence supporting a nuanced, individualized approach to HRT in postmenopausal women. By integrating mechanistic understanding, clinical trial data, observational evidence, and evolving guidelines, clinicians can engage in informed shared decision-making that balances symptom management, quality of life optimization, and cardiovascular risk mitigation for each individual patient.
